# Attitudes of older people with mild dementia and mild cognitive impairment and their relatives about falls risk and prevention: A qualitative study

**DOI:** 10.1371/journal.pone.0177530

**Published:** 2017-05-19

**Authors:** Tamsin Peach, Kristian Pollock, Veronika van der Wardt, Roshan das Nair, Pip Logan, Rowan H Harwood

**Affiliations:** 1 Nottingham CityCare Partnership, Nottingham, United Kingdom; 2 School of Health Sciences, University of Nottingham, Queen's Medical Centre, Nottingham, United Kingdom; 3 Division of Rehabilitation and Ageing, School of Medicine, University Nottingham, Nottingham, United Kingdom; 4 Division of Psychiatry and Applied Psychology, School of Medicine, University of Nottingham, Nottingham, United Kingdom; 5 Division of Rehabilitation and Ageing, School of Medicine, University of Nottingham, Nottingham, United Kingdom; 6 Division of Rehabilitation and Ageing, School of Medicine, University of Nottingham, Nottingham, United Kingdom; 7 Health Care of Older People, Nottingham University Hospitals NHS Trust, Nottingham, United Kingdom; Nathan S Kline Institute, UNITED STATES

## Abstract

**Objective:**

To explore the perceptions of older people with mild dementia and mild cognitive impairment, and their family carers, about falling, falls risk and the acceptability of falls prevention interventions.

**Design:**

Qualitative study involving thematic analysis of semi-structured interviews with patient and relative dyads.

**Participants and setting:**

20 patient/ relative dyads recruited from Memory Assessment Services and Falls Prevention Services in the United Kingdom.

**Results:**

The findings are presented under four key themes: attitudes to falls, attitudes to falls prevention interventions, barriers and facilitators, and the role of relatives. Participants’ attitudes to falls interventions were varied and sometimes conflicting. Some worried about falls, but many resisted identifying themselves as potential ‘fallers’, even despite having fallen, and rejected the idea of needing the help that structured interventions signify. Participants preferred to focus on coping in the present rather than anticipating, and preparing for, an uncertain future. Falls prevention interventions were acknowledged to be valuable in principle and if required in the future but often felt to be not necessary or appropriate at present.

**Conclusions:**

This study of how persons with cognitive impairment, and their relatives, view falls risk and prevention mirror findings relating to the wider population of older persons without dementia. Participants did not generally see falls prevention interventions as currently relevant to themselves. The challenge for clinicians is how to present interventions with understanding and respect for the older person’s identity. They must identify and address goals that patients and relatives value. Simplistic or paternalistic approaches will likely fail. Individualised interventions which focus on maintaining independence and preserving quality of life are more likely to be acceptable by supporting a positive self-image for patients and their relatives.

## Introduction

A fall is defined as unintentionally coming to rest on the ground or at a lower level through whatever cause [1]. Falls, and their consequences of injury, distress, pain, reduced independence, increased anxiety and negative impact on quality of life, are of great significance for older individuals [2], their carers [3–5] and national health care services [6, 7]. Thirty percent of those aged 65 and over, and 50% of those over 80, fall at least once a year [6]. Within the United Kingdom [UK], falls are estimated to cost the National Health Service [NHS] more than £2 billion annually [6, 7]. Dementia causes loss of memory and other cognitive abilities including ‘executive function’ [planning, judgement and decision-making] [8]. Dementia is progressive and irreversible, and interferes with daily activity. ‘Mild cognitive impairment’ [MCI] is a measurable loss of mental function, which does not interfere with daily activities, but often precedes future deterioration. Half of those with MCI subsequently develop dementia [9**]**. There is increasing evidence of the adverse effect of cognitive impairment on balance and increasing falls risk [10, 11] especially executive function [12] and visuospatial problems [13]. A two-fold increased falls risk is present in even mild impairment [14**]**.

Dementia is an increasing public health problem for developed societies; prevalence is strongly associated with age and numbers will double over coming decades [15, 16] There is an urgent need to identify interventions that reduce or delay the dependency resulting from dementia; reducing the risk of falls provides one approach to doing this. Falls prevention interventions in general older populations are limited by low uptake [17, 18] poor knowledge, competing demands on time, perceived lack of benefits, and ill-health [19, 20]. Uptake also depends on health professionals’ attitudes to falls interventions [21] and the older person’s personality [22]. Health professionals commonly believe that falls prevention interventions are ineffective for those with cognitive impairment, although the evidence base is weak [23]. Much previous research has excluded older people with dementia [24, 25].

Older adults may cope with negative conceptions of aging by self-protective behaviour, preserving their identity as capable and ‘still young at heart’ [18, 26]. Falls prevention advice may be seen as threatening and unwelcome [18, 20]. Exercise programmes for people with cognitive impairment have, however, been found to improve strength, function and mood [27–29] and to reduce the risk of falls [30]. In addition, there is evidence in general populations to support wider benefits such as cardio-vascular health and improving participation in social activities [31]. Helping older adults engage in any intervention whilst minimising damage to their positive perception of self is an important but difficult challenge. Family members and relatives play an essential role in supporting rehabilitation and falls prevention interventions in those with cognitive impairments, and their views should be heard when considering the acceptability of services [4, 32].

This paper presents findings from a UK study which explored the views and experiences of people with mild cognitive impairment [MCI] and mild dementia, and their relatives, about falls and what they might do to prevent them. We also explored participants’ views about the facilitators and barriers to professional intervention, and their experiences and thoughts about receiving professional support. This was undertaken to inform the design of an exercise-based fall prevention programme for older people with cognitive impairment or mild dementia. The rationale was to target a group who were still relatively able, but who were at high risk of functional deterioration.

## Method

This research was reviewed and approved by the NHS National Research Ethics Service Committee, East Midlands Ref 13/EM/1061. All participants provided written consent to take part in the study.

People aged 65 years or older with MCI or mild dementia [Mini Mental State Examination 21-26/30 [33], Montreal Cognitive Assessment 15-25/30 [34], depending on the clinical assessment used by recruiting services], and their relatives were recruited to a study of gait and neuro-cognitive risk factors for falls from Memory Clinics or Falls Prevention Services. A convenience sample of twenty patient/relative dyads was recruited to the qualitative study reported here. Dyads who indicated their agreement to take part in a research interview about their experience of falls and perspectives on strategies for prevention were subsequently contacted by a researcher [TP], a falls specialist occupational therapist who was not involved in the participants’ clinical care. Interviews were held jointly with patients and relatives together to gather data on their experience and perspectives on falls and falls prevention, and on relatives’ role in supporting patients to avoid falls.

All participants were deemed to have mental capacity to consent by the recruiting clinician following a structured assessment, and all provided written consent to take part. The study was approved by an NHS Research Ethics Committee.

### Data collection

#### Participants

Twenty interviews were completed with 20 patients and 21 relatives. Sixteen patients were recruited from memory clinics and four from falls prevention services in the UK between 2013 and 2014. One patient forgot that a relative should be present so was interviewed alone, and two interviews were completed with two relatives present. At recruitment, eight patients had an established diagnosis of dementia. The remaining twelve had completed assessments at the memory clinic and were awaiting diagnosis. The relatives interviewed in this study fitted the definition of a carer as: ‘anyone…. who looks after a family member, partner or friend who needs help because of their illness, frailty, disability a mental health problem or an addiction and cannot cope without their support. The care they give is unpaid.’ [https://www.england.nhs.uk/commissioning/comm-carers/carer-facts/].

However, given the early stage of patients’ cognitive impairment not all relatives needed to provide practical assistance, other than the help and support that exists in a caring relationship. To reflect this, the word ‘relative’ is used to refer to the spouses, children and friends of patients participating in the study. For clarity, as both those with early dementia and their relatives were participants in the study, they will be referred to as ‘patient’ or ‘person with cognitive impairment;’ and ‘relative’, rather than ‘participant’, unless both relatives and patients are being referred to, when ‘participant’ will be used.

Semi-structured interviews were conducted in participants’ homes using an interview guide. Interviews were audio recorded, and transcribed verbatim for thematic analysis. All interviews explored attitudes to specific falls prevention interventions [exercise, adaptations, mobility aids and group activities]. The interview also covered experience of falls, current activity limitation, concerns about the future and perceived barriers and facilitators to acceptance of falls prevention interventions. Interviews with cognitively impaired patients have challenges including attention and concentration lapses, problems with memory, abstract thinking and reasoning, word finding difficulties, fatigue, anxiety, and repetition [35]. These were managed with attention to communication strategies such as simplifying question structure and giving plenty of time for responses.

#### Data analysis

A thematic analysis was undertaken using the qualitative software analysis programme NVIVO 10 to manage the data [36]. All transcripts were coded by TP using a process of constant comparison. Eight transcripts were also independently read and analysed by KP as the coding frame was developed through a process of collaborative reflection and discussion and then applied to all transcripts [37, 38]. Coding extended beyond the topics underpinning the semi-structured interviews to incorporate data-driven themes around lifestyle and identity which emerged in addition. Coding of all transcripts was reviewed and the analysis developed by TP and KP following completion of the initial analysis and in the light of themes and patterns that emerged throughout the interviews.

### Findings

Patients were aged between 70 and 93 years and included 13 men and seven women. All but one identified as white British. Most relatives were co-resident spouses [n = 10], or children [n = 9] of the person with cognitive impairment, who did not live with the patient. In addition, one friend and one grandchild took part.

Findings are presented under four key themes: attitudes to falls, attitudes to falls prevention interventions, barriers and facilitators, and the role of relatives. Pseudonyms have been used throughout.

### Attitudes to falls

Thirteen patients reported that they had previously fallen at least once, and gave accounts of their falls, often describing injuries. It should be noted that these reports relate to *accounts* of falls which respondents remembered and were willing to acknowledge, rather than a record of ‘facts’ regarding these experiences. In addition, patients may have been operating with different assessments of what constituted a ‘fall’, especially when they were anxious to resist the attribution of 'someone who falls'. For example, interviews with three of the seven patients who stated that they had not fallen included descriptions of incidents [given by the patient or relative] which seemed likely to have constituted falls from a professional perspective. Participants' accounts referred to falls that had occurred, sometimes recurrently, between 30 years and six months previously. Some respondents could not recall exactly when they had fallen, but most of those who acknowledged having fallen described experience of falls within the last two years. The shock of falling was discussed, and its impact on confidence. In such cases, fear was expressed about the risk of hurting themselves, not being able to get up, or having to move into a residential care or nursing home. Relatives reported that they had to modify their own activities and increase their supervision of the patient because of the risk of falls. One relative, who had fallen herself, said that falls were the ‘worst thing that can happen’; a reminder that the patient is not the only vulnerable person in the dyad. Some relatives were more nervous of the patient falling than the individual was themselves.

It’s getting a little bit scary for us as well to take her out.

Mrs Simmonds’ son

I think we’re getting to a stage where we’re more worried about what if there was a fall whilst she was out.

Mrs Jones’ daughter

When patients could not fully recall their falls, relatives supplied details to complete their account. In one case the relative supplied the full account as the patient was unable to remember any details. Reasons given for falling were environmental [for example tripping on uneven pavements, slipping on wet leaves], or linked to unfamiliarity with the environment. Some falls were attributed to personal limitations or carelessness [‘my own stupid fault’], others to chance or arbitrary mishap. For all participants, both those with experience of a fall and those without, there was a low level of awareness of possible reasons for falls; only poor eyesight and uneven surfaces or ‘catching your feet’ were mentioned. The importance of muscle strength and good balance in reducing the risk of a falls was not widely acknowledged.

Several patients, all of whom had experience of falling, reported being wary or fearful of falling, describing this awareness as being ‘always in my mind’ or ‘watching the entire time’. The others reported no specific fear of falling. Some acknowledged an awareness of the risk of falling but denied that this caused anxiety. Most patients considered that removing or avoiding the identified hazard, or increasing vigilance [‘being careful’] was sufficient to reduce their risk.

*It’s always got to be something at the back of your mind that, you know, you must never forget*.

Mr Brown

*And I don’t think he does worry about it, because to worry about it restricts him*.

Mr Taylor’s friend

*No, not while I’m all right and walking about, no, it doesn’t even occur to me that I might fall, never even thought about it*.

Mrs Evans

Patients reported modifying their behaviour [doing activities with people rather than alone, reducing bending, ‘allowing’ for hazards], or exercising caution in specific situations [climbing stairs, crossing roads]. By attributing falls to arbitrary accidents [which could happen to anyone] or carelessness [‘my own silly fault’] they felt that future falls were avoidable by ‘being sensible’. Others who had fallen felt the cause or presumed explanation for their fall had been addressed, thus further intervention was not important. ‘Personality’ was sometimes considered to be a factor in anxiety around falling [being a ‘born worrier’ rather than someone who ‘never worried’], rather than objective risk.

### Attitudes to falls prevention interventions

Participants were asked about their attitudes to health service falls prevention interventions. Views varied from ‘being prepared to try' or 'go along with it’, to obvious reluctance. One dyad had ‘never thought about it’. Recurring responses were: we ‘have it already’, ‘do not need it’, ‘do not want it’ or ‘do not need it at the moment’. Interventions were often seen as good for ‘later on in life’, and ‘out there if needed’, but not necessary at present. Many of the participants reported that they were satisfied with their present situation and therefore felt they did not need interventions. The view that ‘we’re all right, not lacking in anything’ was often expressed. Seeking help ‘would have to be something to do with 'having fallen’, and considered appropriately triggered by a crisis, ‘if needs must’. Even those who were worried about falling did not feel the need to take preventative action in advance of a crisis or initiate this pre-emptively when well.

#### Equipment, adaptations and mobility aids

Participants had access to a wide range of equipment or adaptations [e.g. toilet frame, delta walker, rambler trolley, walking sticks, perching stool, bath board, bed grab handle, grab rails or wet room]. Some had found these very helpful and felt they had ‘undoubtedly saved a lot of falls’, whereas others used them ‘as needed’, or had discontinued their use. Some participants reported contradictory stances. For example, two dyads who had equipment which they said they were using also reported that they did not need it at present. This may reflect a desire to maintain a stance of not needing help, while nevertheless accepting it. It is possible that respondents had normalised the use of these aids and no longer saw them as an active intervention. Equipment was acceptable ‘if needed’, when [in principle] there was thought to be ‘nothing wrong with it’, although there was concern that using such aids was a clear and unwelcome advertisement of vulnerability.

*If it was enabling me to do something that I couldn’t, but wanted to do, I would go along with it*.

Mr Davies

*I mean, I've got no worries about what people might think or not think. I mean, if you need something, if it's going to make your life tolerable shall we say, well, by all means and it's on offer, get it*.

Mr Brown

*One object[ion] I would have to.… I suppose… if it was outside, not because of the look of it, but because it would make strangers aware…what sort of person lived in that property and therefore, they’d be more vulnerable*.

Mr Banks’ wife

Many patients possessed a walking stick, which were reported to be used to variable extents: sticks gave confidence, reassurance or were something to rely on for those using them. However, some patients were openly ambivalent or evasive about the extent to which they used a stick. Others openly rejected the need or were not prepared to use them. Two patients used a delta walker for outdoors and a walking frame for indoors respectively. One relative said they would buy items privately rather than ask for professional help.

*I think, well, if that time came, obviously, I would use it. Yeah, and not averse to using it. I just don’t, I don’t think I need it at the moment*.

Mrs Smith

Mrs Evans’s Daughter: But she doesn’t use a walking stick, anything like that because, you know, she won’t, well, you see it all as a sign of weakness anyway, don’t you?

*Mrs Evans: No, I don’t, no, not a sign of weakness but it hasn’t come to me [the need to use a stick yet]*.

#### Physical exercises

Participants said they were receptive to taking exercise specifically to prevent falls in principle ‘if I thought it was doing me good’, but felt they were not yet, or were no longer, in need. Previously practiced exercises were recognised to have been beneficial, but were discontinued once the need had passed, or only practised occasionally, as the habit had ‘just drifted off somewhere’ or exercise sheets were ‘filed away’. Participants reported that they were currently ‘doing enough’ exercise through ‘normal’ activity such as gardening, walking, and even ‘going to the loo’.

I do do some exercises and I’ve often said to P., ‘Come on, let’s do some’, but you can’t really. Even those later lots you were given from the doctor, when you said your back was bad, and that’s only a few months ago, you didn’t really get yourself going on them, did you?

Mr Davies’ wife

*Well, to be really honest, I feel as though I’m doing enough. I mean I don’t sit all day*.

Mrs Peters

*When I’ve been in the garden, next door- my son lives next door—I was out there doing the lawn and gardening and digging…I come in and I’ve, oh, I’ve done enough exercise, I don’t need to do them*.

Mr Appleby

Participants referred to engaging in a wide range of leisure activities and hobbies which often involved activity and social engagement [including bowls, visiting friends and family, trips out, holidays, sewing, gardening, golf, walking, doing crosswords and puzzles, going into town, bus trips, reading, dancing, church-going]. Continued ability to carry out normal activities of daily living [e.g. cooking, making tea, cooking meals, ironing, housework/hoovering, washing pots] was an important indicator of functional independence [‘we’re alright’]. Alongside assertions of ongoing independence and functional capacity, however, were many statements acknowledging attrition: a giving up or reduction of competencies and activities [no longer dancing, going on holiday, visiting friends and family, walking less far than formerly]. In this case, participants resorted to a strategy of absorbing and normalising loses to redefine themselves as maintaining [sufficient] independence and autonomy [‘we’re alright’].

Many practical challenges for completing prescribed exercises were reported. It was ‘difficult to do the same thing every day’, the exercises given by a physiotherapist were felt to be ‘too difficult’, ‘too much’, ‘too fast’ ‘had not been demonstrated’, or were not seen as relevant. Participants also reported that exercise may require family supervision, were difficult to fit into the day, or that it was difficult to motivate oneself to do. They were put off by not knowing where to access appropriate exercise in the community, or because they felt awkward and out of place in public places: e.g. feeling ‘like an old lady’ in the gym.

Some participants expressed an intention to start exercising again but did not report concrete plans for doing so. Several relatives described trying to prompt patients to exercise, but without success. Indeed, the need to remind patients, or monitor their exercise engagement, could constitute an unwelcome obligation. No relatives reported seeking to find or implement an exercise regime that they could do daily with the patient.

#### Group activity interventions

Three broad responses were expressed towards group activities: positive, negative, and those who were not averse in principle but were not likely to take part in practice. Several patients reported currently attending existing community social group and being happy with these. Others reported that they would join a group if invited, particularly if this involved their own age group and similar people ‘right in my bracket’. They felt they would feel more stretched and motivated to exercise, and would be happy to join in. They stated they would enjoy the social element and getting out of the house ['better to go somewhere'] and even if the group was ‘boring’ it was still ‘something to do’. Community venues were considered acceptable provided they had the right skills to manage people, and were in an appropriate location. Relatives liked the idea of a group as this could give them respite from their caring role, although they respected the patients’ preferences in this.

*I wouldn’t mind attending a clinic if, if it was, you know, somewhere I could get to, taking part in activities with other old people*.

Mr Taylor

*I’d rather do it in a, you know, in a group…Yeah. With others in the same boat*.

Mrs Walker

However, a third of patients said that they would not be interested in joining a group because they considered these would not be helpful, and anticipated that they would find them patronising, boring or a waste of time. Concern was expressed at groups not being geared for older people or not adapted to the participants’ levels of need.

It doesn’t appeal to me. Not because I’m sort of stand-offish or anything else, I think I’d be bored…That sounds rather pompous and I don’t mean it to sound that…

Mrs Thompson

One patient felt that a group would show that she was ‘not capable of being on my own and doing something on my own’. Some implied that they ‘can’t face Alzheimer’s groups’ as they feared these would underline the potential progression of the disease. Another view was that the respondent was in a ‘different league’ from those for whom group activities were appropriate, being ‘top of the class’ or ‘not bad enough’ compared to others attending. Some patients were concerned that they might not know anyone or felt that they were simply ‘not group people’ no matter what the group.

### Barriers and facilitators to exercise

Several barriers to activity or exercise were mentioned in the interviews by both patients and relatives. The challenges for this group were public [services, location, environment, transport]; extrinsic [cost, time] and intrinsic [health, emotions, motivation]. Throughout the interviews it became clear that cognitive impairment presented challenges, such as route-finding, motivation and remembering appointments, although this was expressed in general conversation rather than overtly acknowledged as a barrier. It is simplistic to separate these factors due to the complex interrelationships existing between them. Facilitators to interventions were usually the inverse of the barrier [i.e., bad health is a barrier; good health a facilitator], but were not clearly articulated by participants. Support and supervision when completing exercises were considered important to successful intervention as was establishing the right level and relevance of interventions for people with cognitive impairment.

### Relatives' role

Patients in this study were in the early stages of dementia and were reported to be independent in most activities of daily living [ADLs]. The relative who was interviewed did not necessarily define themselves as a ‘carer’, although some acknowledged this to be an increasing role. Relatives expressed wanting to provide practical support whilst not undermining the patient’s independence. Some relatives reported that they provided reassurance and guidance with daily activities, or undertook monitoring of activities of the patient to promote their safety. Relatives varied widely in their ability, resources and motivation to provide such support, which even when given willingly was reported to be exhausting and demanding. One relative described the fatigue resulting from her caring role. She reported that clinician expectations of her support for her husband were ‘just one thing when you’ve got everything else’.

Not that, I’d not stop her but I’d make sure that I…. took her or, you know, and collected her…

Mrs Walker's Daughter

*I don’t know, you’d just have to see how things change, if they get worse and then you’re struggling, and then there’s help there, then obviously you would ask. But in between we try and look after mum best we can, and we do more and more slowly, you know, it builds up on you, you just do more and more for her than you used to. But as long as it’s acceptable then you carry on*.

Mrs Simmond's son

Family dynamics affected the nature of the support provided by the relatives—e.g. whether relationships involved a couple ‘living for each other’, or a son or daughter not wanting to ‘push’ their mother into challenging activities, or establishing a rota of family members to provide support. Relatives acknowledged their limits; e.g. feeling that they ‘can’t pick them up’, or ‘don’t know how to handle it’. Some had experienced role reversals and a change in the domestic division of labour resulting from patient’s cognitive decline. Other relatives acknowledged the symbiotic nature of the relationship: ‘I need your strength, [while you need my memory]'. Availability and access to relatives were acknowledged as factors affecting the support that could be given. Patients did not want to rely on family as ‘they have their own lives’, and could feel guilty about the restrictions which their illness placed onto their relative on whom they strongly did not wish to impose a burden. Both patients and their relatives also had a range of competing obligations and commitments; to spouses, children, grandchildren or even dogs. However, it was notable that even in relation to those with early dementia or mild cognitive impairment, relatives tended to speak for the patient in the interviews. This was often encouraged by the patient: ‘I’m looking at you [to answer]’; ‘I think your thoughts are more appropriate’. This contribution came across as a loving support or simple reminder, rather than controlling or speaking over.

## Discussion

This study expands our knowledge of patient and relatives’ attitudes to falls risk in the early stages of cognitive impairment and moves towards an understanding of the challenges in engaging this group to maintain health and prevent future falls. Most patients were aware of the risk of falls. A minority were keen to intervene to reduce risk. A quarter of patients reported being engaged in some kind of community group exercise, but a third indicated no interest in this kind of activity now or in the future. Overall, however, patients expressed being open to falls prevention interventions in principle and in the future, but tended to present themselves as ‘doing alright’ and not in need of such measures at the present time. They held different preferences regarding falls interventions which might subsequently be appropriate, and varied in their receptiveness to information and falls prevention strategies; what was a solution for one was not acceptable to another. Numerous barriers to exercise interventions were cited. Supervision was considered important, but relatives were mixed in their willingness and ability to provide this. Individual circumstances and relationships were very important in determining what might be possible and acceptable.

The semi-structured interview approach allowed an in-depth exploration of views, including topics not anticipated in advance. Interviewing twenty dyads enabled a wide range of views to be obtained. By interviewing dyads, the person with cognitive impairment was supported, and their relative was also encouraged to have a voice and make a valued contribution to the study. Accounts were retrospective, so details may have been forgotten, recalled selectively, or issues with insight or judgement may have influenced what was said, such as the tendency to discount or re-define non-injurious falls. It is likely that the incidence of falls, slips or trips was underreported. Relatives may have been selective in what they said in the presence of the person with cognitive impairment, and manifest a desire to articulate their own needs, or psychological defences to facing an uncertain future. The participants were volunteers, predominantly white and reasonably affluent. Different perspectives may have been articulated by a more diverse sample and the findings of this exploratory qualitative study may not be generalised to wider populations. However, their resonance with previous research findings reinforces their relevance and theoretical transferability to other settings [20, 39].

Few previous studies have explored the views of people with cognitive impairment and their relatives about exercise and falls prevention [32], but our findings are similar to those reported for populations without cognitive impairment [4, 18, 20, 22, 39, 40]. Dickenson et. al. [19] describe knowledge, availability, appropriate facilities and design, and experiencing benefits as key facilitators for engaging in falls prevention interventions. Other work with people with dementia [41] indicated that ensuring activities are pitched at the correct level is key to engagement. Health professionals and their response to reported falls played a major role in referral to, and uptake of, interventions [42] as did other forms of positive social reinforcement [23]. Issues such as lack of time and money, and accessibility of location, knowledge about and availability of appropriate services and motivation are barriers to exercise among the general population as well as those affected by cognitive impairment [43, 44]. In maintaining their lives at home, our participants demonstrated resilience and ability, and like frailer people more generally, ‘balance[d] loss and capacity in their everyday lives’ [45]. Participants expressed the hope that they would remain as they were, linking with De Witt et al’s [46] concept of people with dementia ‘holding back time’. The view that ‘anyone can fall’ or suffer an accident, minimises the link to personal vulnerability [18]. Participants resisted a sense of vulnerability and also acknowledged it; the same individual could do both in different parts of the interview, illustrating the tension between the 'real' and the 'ideal' or 'private' and 'public' accounts [47].

Studies have found that relatives are important in ensuring the success of interventions, for positive social reinforcement, as well as providing practical support in matters such as transport [32]. Relatives often act as activity enablers whilst also gate keeping to protect their loved one from potentially demanding or hazardous situations. Relatives have an important role in supporting patients and their input can be critical to the success of effective falls intervention [32]. However, they experience difficulties and ambivalence towards providing this, especially as the patient’s conditions deteriorates [3–5, 48].

The idea that ‘things are OK’ might spring from the desire to maintain a positive sense of identity. This may involve redefining what ‘OK’ is for that person–‘managing’ or ‘soldiering on’ in preserving independence are seen as virtues [18, 22, 39]. Identifying with the current relevance of interventions is a critical point, as anticipation of the future, including the goal of preventing future decline, is not always a cause of present motivation [20]. Participants described making changes to their lives in response to their deteriorating abilities. This contrasts with their expressed idea that interventions were not currently relevant. Indeed, the ‘subjective perception of risk is often at odds with the objective benefits of the behaviour’ [43]. Thus, participants balanced the risk of acknowledging themselves as weaker, and a potential faller, against the potential benefits of falls prevention interventions. Being in need of help is highly disvalued [49] and participants did not present themselves as in need of help during the interviews, preferring to retain a capable presentation of self: ‘we’re doing alright’ [40].

Age-related conditions combined with deteriorating memory are additional challenges for those affected by cognitive impairment [50], particularly the very old: several of our patients were over 90. Co-morbidity and age impacted on patients’ ability to manage physical and social environments, forcing them to adapt or avoid environments that were too challenging. Diminishing cognition was not an openly acknowledged barrier for engaging in interventions, although this would potentially present problems [e.g. finding a new location, remembering appointments, learning new skills], especially without carer support. However, while this is an understandable strategy, risk aversion and using ‘being careful’ as a primary coping mechanism can result in deskilling and the individual’s loss of confidence in their capacity to undertake activities of daily living [20].

Participants who expressed increasing caution felt they reduced their hypothetical risk of falls because they were careful, and so did not perceive themselves at risk of falling. Even those who had fallen, because they were now careful in the situation they attributed as the cause of their fall, did not report themselves to be at risk of future falls [18, 51]. Indeed, the participants interviewed were largely independent and working to maintain function in their own ways. However, the introduction of effective exercise interventions at this point could be critical in enabling people with cognitive impairment [and their relatives] to develop and maintain key skills and prolong functional capacity and sustain their independence in the future.

Slowing or reversing decline through exercise and increased activity could help to preserve the independence and functional capacity which are important constituents of good quality of life [52]. Although perceived risk of falling did change behaviour among some participants, the importance of improving strength and balance to reduce the risk of falls in future was not generally appreciated. This reflects previous findings and demonstrates that this is still an area with great scope for awareness-changing within the general population [18, 20, 39].

It is also important to consider the role of relatives as potentially a barrier as well as a facilitator to successful interventions; they may lack the time or willingness to support the person with cognitive impairment with the intervention, or lack the energy or physical capability required to do this.

### Implications

To engage patients with mild dementia or mild MCI in effective falls prevention we must reach agreement on goals that are important and meaningful to the individuals concerned. There is a delicate balance to be maintained between supporting patients’ desire to maintain their ongoing integrity as independent agents who are managing to ‘do alright', and generating awareness of their current and future risk. The goal is to engender acceptance of the value of falls interventions as effective measures to protect or even increase wellbeing and independence, rather than being viewed as a public and personal signifier of frailty and incapacity. Recruitment messages that emphasize the multiple benefits of interventions such as enjoyment, health maintenance, improving balance, retaining mobility and independence may have more relevance to older people’s motivation to engage with interventions than directly emphasising the risk of falls [22, 32, 39, 50]. Falls prevention strategies of compensation, rehabilitation and education need to be personalised to be effective [52]. Interventions should be framed in a way that positively appeals to the individual and move beyond the rhetoric of ‘tailoring’ [50]. Services need to focus on the scope for such interventions to contribute to both personal and family wellbeing, as well as relieving economic and demand burden on health care services.

Relatives or other caregivers are often critical to successful exercise engagement of patients with dementia. Understandably, however, they may not relish the role of being the person providing the intervention alongside balancing a range of other responsibilities and commitments. It should be noted, also, that relatives are often old and frail themselves. Becoming the ‘personal trainer’ for their loved one may not be their priority or within their capacity. This has an implication for practice as long-term continuation of exercise programmes may often have to rely on continuing professional or group support, rather than be delegated to informal care. An individually adapted approach for couples, which values the role of the carer and accounts for the progressive and changing nature of dementia, should be a guiding principle for intervention design. If an intervention is being promoted as good for the patient’s health and wellbeing, it would seem wise to promote it to the relative too.

Participants expressed complex, inconsistent and contradictory positions on falls risk, their current abilities and need for interventions. Part of the challenge for clinicians is to find ways to bridge these gaps and work with people with cognitive impairments and their relatives to fit solutions into their existing lives and abilities. Further work is needed to explore practical ways of achieving this.

## Conclusion

This study builds on the existing literature by finding that people with cognitive impairment and their relatives are similar to the wider population of older people in expressing themselves to be amenable, in principle, to falls prevention intervention, but not much interested in practice at the present time. They will need support to personally identify their current relevance and future gain. The introduction of therapeutic interventions, with their consequent implication of diminished capacity, needs to be balanced with preserving confidence and a positive sense of self. Clinicians need to focus on promoting present health, methods to preserve quality of life, independence and wellbeing, as opposed to talking about falls prevention and the negative prospect of risk in order to improve uptake. Pragmatic barriers that everyone faces must also be overcome to ensure accessibility of interventions. Health professionals need to take up the challenge of motivating a group of people with complex needs, who require input to remain independent and preserve quality of life as long as possible.
